# Clinical characteristics and risk factors of atrial fibrillation detection after acute ischemic stroke

**DOI:** 10.3389/fcvm.2026.1836796

**Published:** 2026-07-08

**Authors:** Hui Zhao, Yingyu Jiang, Li Liu, Jingjing Li, Jinxin Shi, Weijia Wang, Xiaoling Cheng

**Affiliations:** 1 Department of Internal Medicine, Beijing Tiantan Hospital, Capital Medical University, Beijing, China; 2 China National Clinical Research Center for Neurological Disease, Beijing Tiantan Hospital, Capital Medical University, China

**Keywords:** atrial fibrillation, predictors, recurrence, risk factors, stroke

## Abstract

**Background:**

The atrial fibrillation detected after stroke (AFDAS) confers an elevated risk of unfavorable outcomes for acute ischemic stroke (AIS) patients, necessitating early identification of high-risk patients.

**Method:**

This study was a secondary analysis of data from the prospective, multicenter China National Stroke Registry II (CNSR-II). AFDAS was defined as a new atrial fibrillation episode (> 30 s) detected after the onset of AIS. Patients who had AFDAS but without prior atrial fibrillation history were included in this study. Multivariable logistic regression was used to identify risk factors linked to AFDAS. All patients were followed for 1 year to assess stroke recurrence. And then we explored the relationship between AFDAS and ischemic stroke recurrence by using multivariable logistic regression analysis.

**Results:**

Among 23,446 patients, 572 (2.4%) developed AFDAS. These patients were older and had more severe neurological deficits on admission than those who remained in sinus rhythm. Independent risk factors for AFDAS included older age (≥ 65 years: OR = 2.14, 95% CI: 1.44–3.16), history of coronary arterial disease (OR = 1.88, 95% CI: 1.22–2.92), elevated C-reactive protein (OR = 1.02, 95% CI: 1.01–1.02), and lower triglyceride levels (OR = 0.76, 95% CI: 0.61–0.96). During a 1-year follow-up, recurrent stroke occurred in 1,467 patients (6.3%). The recurrence rate was significantly higher in the AFDAS group than in the SR group (11.7% vs. 6.1%, *P* < 0.001). AFDAS was more prevalent in patients with recurrence (4.6% vs. 2.3%, *P* < 0.001) and remained an independent risk factor for 1-year stroke recurrence after adjusting for traditional risk factors (OR = 1.82, *P* < 0.001).

**Conclusions:**

Older age, coronary heart disease history, and inflammatory marker (CRP) may assist in early identification of AFDAS, a condition associated with significantly increased stroke recurrence risk. Targeted monitoring and secondary prevention strategies are warranted, especially for AFDAS patients with prior cerebrovascular or cardiac comorbidities.

## 1 Introduction

Stroke remains the most important cause of permanent disabilities and cognitive deficits, accounting for approximately 5.2% of all deaths worldwide (1). Acute ischemic stroke (AIS) is a medical emergency caused by the transient or permanent occlusion of cerebral vessels, which constitutes the majority of strokes (2). Atrial fibrillation (AF) is identified as a powerful contributor to incident ischemic stroke, increasing the risk by five to six times without influencing other risk variables (3). In recent years, growing evidence indicates that a substantial proportion of AIS patients without a prior history of AF develop newly detected AF after the index stroke, with reported incidence rates ranging from 4% to 24% (4–6). This phenomenon, referred to as atrial fibrillation detected after stroke (AFDAS), has been correlated with adverse outcomes, including an increased risk of recurrent stroke and mortality (7–9). Importantly, emerging studies suggest that AFDAS may differ from pre-existing AF in terms of pathogenesis, clinical course, and prognostic implications, underscoring the need to recognize AFDAS as a distinct clinical condition that warrants targeted investigation (10, 11).

Early detection of AFDAS is crucial for initiating appropriate anticoagulation therapy, which can reduce the risk of recurrent stroke by up to 60% (12). However, AFDAS is often paroxysmal and presents with short episodes, complicating its clinical detection and potentially resulting in underdiagnosis (11). Although extended cardiac monitoring improves AFDAS identification, its high cost and logistical barriers limit widespread implementation. Moreover, the risk factors and predictors of AFDAS remain inconsistent across studies, and there is a paucity of validated prediction models incorporating readily available clinical and laboratory parameters, particularly in large Asian cohorts (6, 13). Furthermore, whether AFDAS is associated with an increased risk of recurrent stroke remains unclear, which brings uncertainties to secondary prevention strategies (8).

Therefore, this study aimed to: (1) identify and verify independent predictive factors for AFDAS; (2) characterize the clinical features of patients with recurrent stroke and investigate the association between AFDAS and recurrent ischemic stroke. The findings may facilitate early risk stratification and guide tailored monitoring and intervention strategies in patients with AIS.

## 2 Methods

### 2.1 Study population

The study conducted a secondary analysis using the CNSR-II dataset. CNSR-II was a nationwide prospective registry in China and was designed to evaluate stroke characteristics and care delivery in clinical settings. Participants were recruited in sequence from 219 urban hospitals in the CNSR-II between June 2012 and January 2013. They were included if they met the following criteria: (1) aged 18 years or above; (2) diagnosed with ischemic stroke, transient ischemic attack (TIA), spontaneous intracerebral hemorrhage, or subarachnoid hemorrhage within 7 days of the index event; (3) directly admitted to hospital from an outpatient clinic or emergency department; and (4) provided written informed consent by the patient or a legally authorized representative. Detailed protocols and additional information regarding the CNSR-II have been published previously (14, 15).

For this study, strict inclusion and exclusion criteria were applied to define the study cohort based on the original registry population. Inclusion criteria comprised clinically confirmed AIS, absence of prior atrial fibrillation or flutter history, and undergoing at least one ECG or ≥24-hour ECG monitoring during hospitalization. Exclusion criteria included diagnosis of hemorrhagic stroke or brain tumor, in-hospital stroke occurrence, or missing key data for database analysis. All participants provided written informed consent, and ethical approval was obtained from the Central Institutional Review Board of Beijing Tiantan Hospital for this research.

### 2.2 Data collection

Baseline data encompassing demographics, risk factors [prior stroke, hypertension, diabetes, coronary heart disease (CHD), heart failure, peripheral artery disease, and current/previous smoking status], as well as key laboratory parameters (International Normalized Ratio [INR], C-reactive protein [CRP], glycosylated hemoglobin, blood urea nitrogen [BUN], cholesterol [TC], triglyceride [TG], high-density lipoprotein [HDL], low-density lipoprotein [LDL]), were collected for all study participants upon admission, and more than half of the patients presented to the hospital within 24 h after symptom onset. The trained research coordinators at each study center had collected and recorded these data in electronic databases, as detailed in a prior study (16). CHD was defined as a documented prior history of coronary atherosclerosis-induced myocardial ischemic disorders, including stable angina, unstable angina, prior myocardial infarction, or previous coronary revascularization procedures (percutaneous coronary intervention or coronary artery bypass grafting).

Current smoking was defined as habitual cigarette smoking within 6 months before stroke onset at baseline admission. The National Institutes of Health Stroke Scale (NIHSS) was used to systematically assess baseline neurological status within the first twenty-four hours following hospital admission.

A documented history of atrial fibrillation or atrial flutter was confirmed by either at least one electrocardiographic recording or the retrieval of relevant prior medical records. For this study, AFDAS was strictly defined as follows: no prior medical history of AF and confirmation of an AF episode lasting more than 30 s, as evidenced by either continuous electrocardiographic monitoring throughout hospitalization or serial daily 12-lead electrocardiograms obtained subsequent to the index ischemic stroke. AFDAS only refers to new-onset AF episodes detected after AIS onset during in-hospital ECG or at the time of admission.

### 2.3 Follow-up and outcomes

The follow-up procedure of the CNSR-II trial has been published previously in detail (16). The primary outcomes were 1-year ischemic stroke recurrence and all-cause death. Standardized telephone follow-ups were performed at 3, 6, and 12 months by trained researchers from Beijing Tiantan Hospital following fixed interview protocols. Patients served as primary interviewees; caregivers were interviewed if patients were unreachable or provided unreliable information. Recurrent strokes requiring readmission were confirmed via reviewing corresponding hospital medical records. For suspected recurrent events without hospitalization, final diagnostic decisions were reached through joint assessment by research coordinators and the principal investigator.

### 2.4 Statistical analysis

Statistical analyses utilized SPSS Statistics version 25. Continuous variables were presented as mean ± SD or median with IQR, depending on data distribution normality; categorical variables were depicted as frequencies and corresponding percentages. Between-group comparisons [AFDAS group vs. SR (sinus rhythm) group] for continuous variables were executed through the independent-samples *t*-test (for normally distributed data) or Mann–Whitney *U*-test (for non-normally distributed data). Intergroup disparities for categorical variables were evaluated using the chi-square (*χ*^2^) test or Fisher's exact test, as applicable. Univariate and multivariate logistic regression analysis were used to identify independent risk factors for AFDAS. Risk factors for recurrent ischemic stroke were also assessed by using multivariate logistic regression analysis. To clarify the independent association between AFDAS and recurrent stroke, two adjusted models were applied to control confounders. Model 1 was adjusted for demographic features and vascular comorbidities. Model 2 was additionally adjusted for long-term medications based on Model 1. Odds ratios with 95% confidence intervals were calculated, and a two-tailed *P* < 0.05 was considered statistically significant. For laboratory variables with missing values, a complete-case analysis was used in the multivariable regression models (missing value proportions of data about 18.1%). We compared baseline characteristics between participants excluded for missing data and those included in the final analysis. No significant intergroup differences were found in age, sex, and major clinical variables.

## 3 Results

### 3.1 Baseline characteristics

**Figure 1:**
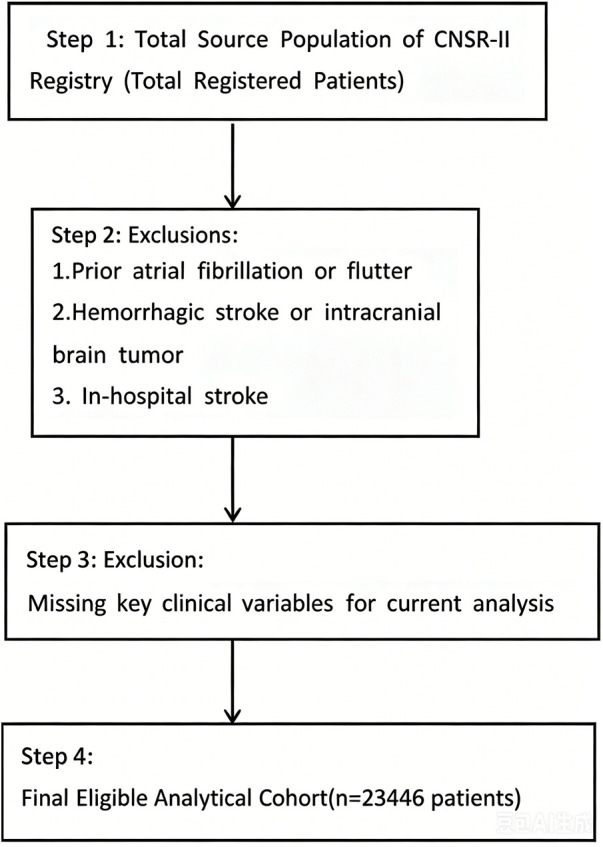
The flowchart of patient enrollment. *Due to the closure of the registry database, the exact number of patients excluded at each step could not be individually retrieved. Only the final analytical cohort size n=23,446 is reported.

As shown in Figure 1, 23,446 patients with AIS were enrolled in this study. The mean age of the study subjects was 63.9 ± 12.1 years old, and 14,836 (63.3%) patients were male. Of these, 572 patients (2.4%) were newly diagnosed with atrial fibrillation during hospitalization and constituted the AFDAS group, while the remaining 22,874 patients (97.6%) maintained SR throughout the monitoring period. As summarized in Table 1, compared to patients in the SR group, those in the AFDAS group were significantly older (69.8 ± 12.7 vs. 63.7 ± 12.0 years, *p* < 0.001) and had a slightly lower BMI (23.8 ± 3.7 vs. 24.1 ± 3.6 kg/m^2^, *p* = 0.025). Neurological deficit on admission, as assessed by the NIHSS, was more severe in the AFDAS group [median [IQR]: 5.0 [2.0–12.0] vs. 3.0 [1.0–7.0], *p* < 0.001]. Regarding medical history, a history of CHD (24.8% vs. 11.0%, *p* < 0.001) and heart failure (0.9% vs. 0.4%, *p* = 0.042) were more prevalent in the AFDAS group. There were no significant between-group differences in the prevalence of hypertension, diabetes mellitus, or prior stroke/TIA (*p* > 0.05 for all).

**Table 1:** Baseline characteristics of included patients.

Clinical variables	Total (*n* = 23,446)	SR (*n* = 22,874)	AFDAS (*n* = 572)	*P*
Age (year), mean ± SD	63.9 ± 12.1	63.7 ± 12.0	69.8 ± 12.7	**<0**.**001**
Female, *n* (%)	8,610 (36.7)	8,370 (36.6)	240 (42.0)	**0**.**009**
BMI (kg/m^2^), mean ± SD	24.1 ± 3.6	24.1 ± 3.6	23.8 ± 3.7	**0**.**025**
Heart rate (beats/min)	75.1 ± 11.4	75.1 ± 11.2	78.1 ± 16.4	**0**.**004**
SBP (mmHg), mean ± SD	150.8 ± 24.3	150.9 ± 24.3	148.2 ± 24.1	**0**.**015**
DBP (mmHg), mean ± SD	88.3 ± 14.1	88.4 ± 14.1	86.9 ± 14.8	0.057
NIHSS score, IQR				**<0**.**001**
Mild (1–4)	14,424 (61.5)	14,157 (61.9)	267 (46.7)	
Moderate (5–15)	7,540 (32.2)	7,329 (32.0)	211 (36.9)	
Moderate/severe (> 20)	1,482 (6.3)	1,388 (6.1)	94 (16.4)	
Medical history				
Hypertension, *n* (%)	15,136 (64.6)	14,766 (64.6)	370 (64.7)	0.948
Diabetes mellitus, *n* (%)	4,398 (18.8)	4,295 (18.8)	103 (18.0)	0.641
Current smoking, *n* (%)	6,887 (29.4)	6,752 (29.5)	135 (23.6)	**0**.**002**
Prior stroke or TIA, *n* (%)	7,152 (30.5)	6,981 (30.5)	171 (29.9)	0.749
Coronary heart disease, *n* (%)	2,652 (11.3)	2,510 (11.0)	142 (24.8)	**<0**.**001**
Heart failure, *n* (%)	86 (0.4)	81 (0.4)	5 (0.9)	**0**.**042**
Peripheral disease, *n* (%)	738 (3.1)	726 (3.2)	12 (2.1)	0.145
Biochemical parameters on admission				
INR, mean ± SD	1.0 ± 0.3	1.0 ± 0.3	1.1 ± 0.6	**<0**.**001**
HbA1C (%), mean ± SD	6.5 ± 1.7	6.5 ± 1.7	6.3 ± 1.4	**0**.**006**
CRP (mg/L), mean ± SD	8.2 ± 13.8	8.0 ± 13.5	13.2 ± 21.2	**0**.**001**
BUN (mmol/L), mean ± SD	5.4 ± 2.1	5.4 ± 2.1	5.9 ± 2.3	**<0**.**001**
TG (mmol/L), mean ± SD	1.7 ± 1.3	1.7 ± 1.3	1.4 ± 1.1	**<0**.**001**
TC (mmol/L), mean ± SD	4.7 ± 1.1	4.7 ± 1.1	4.4 ± 1.1	**<0**.**001**
LDL (mmol/L), mean ± SD	2.8 ± 1.1	2.9 ± 1.0	2.6 ± 0.9	**<0**.**001**
HDL (mmol/L), mean ± SD	1.2 ± 0.4	1.2 ± 0.4	1.2 ± 0.3	0.862

Bold represents significant values where the *p*-value is less than 0.05; AFDAS, atrial fibrillation detected after stroke; BMI, body mass index; BUN, blood urea nitrogen; HbA1C, Glycated Hemoglobin A1C; CRP, c-reactive protein; DBP, diastolic blood pressure; TG, Triglyceride; TC, total cholesterol; HDL, high density protein; LDL, low density protein; IQR, interquartile range; NIHSS, National Institutes of Health Stroke Scale; TIA, transient ischemic attack; SBP, systolic blood pressure; SD, standard deviation; SR, sinus rhythm.

In terms of biochemical parameters on admission, patients with AFDAS had significantly higher serum levels of CRP (13.2 ± 21.2 vs. 8.0 ± 13.5 mg/L, *p* = 0.001) but notably lower TC (4.4 ± 1.1 vs. 4.7 ± 1.1 mmol/L, *p* < 0.001) and TG (1.4 ± 1.1 vs. 1.7 ± 1.3 mmol/L, *p* < 0.001) compared to the SR group.

### 3.2 Analysis of risk factors for AFDAS

**Figure 2:**
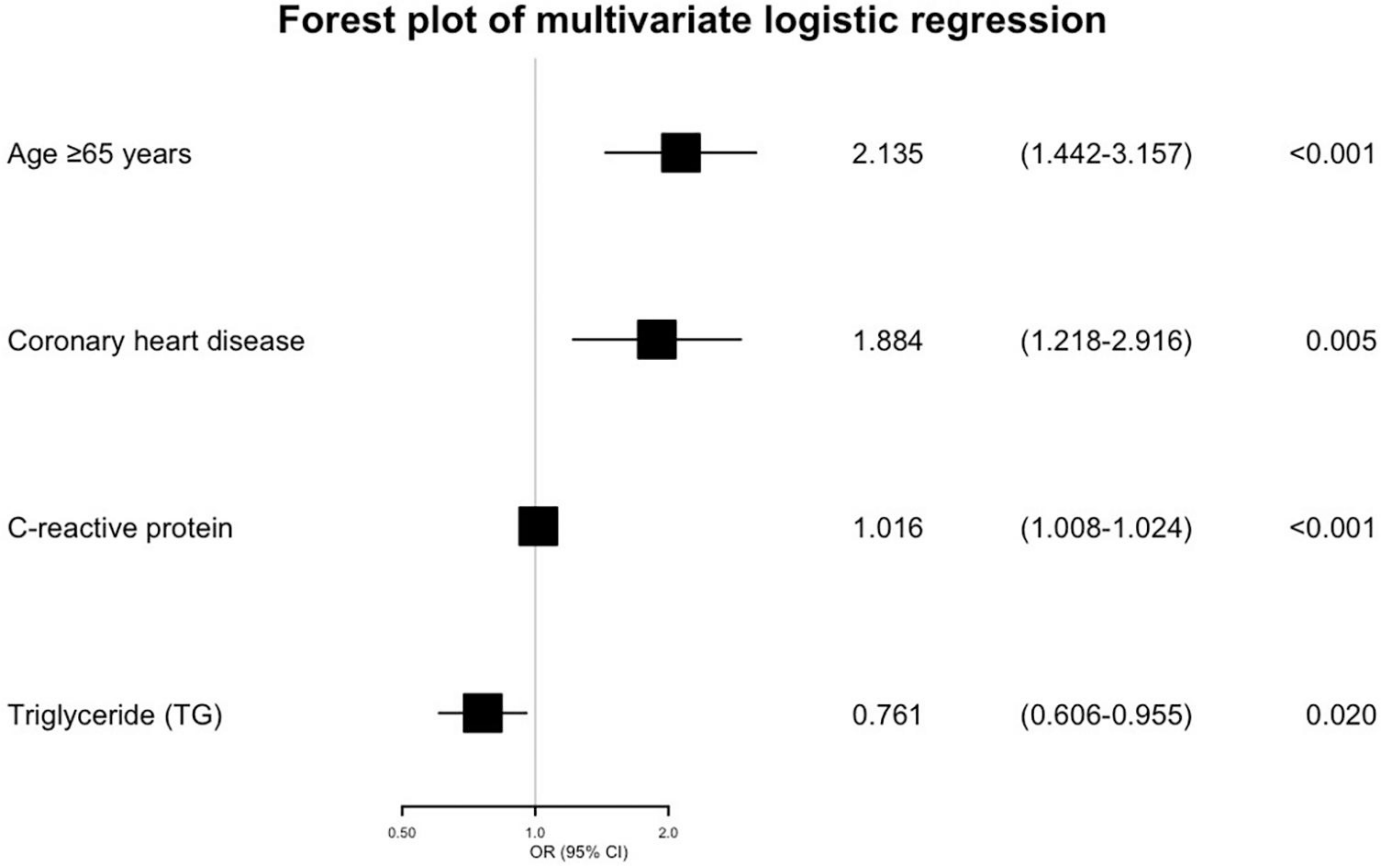
Forest plot of multivariate logistic regression. Forest plot displaying adjusted odds ratios and 95% confidence intervals for independent predictors of AFDAS, including age ≥65 years, history of coronary heart disease, C-reactive protein, and triglyceride levels.

Nine variables significantly correlated with AFDAS were identified through univariate logistic regression analysis (*P* < 0.05): age, BMI, history of CHD, history of heart failure, current smoking, and admission levels of HbA1c, LDL, TG, and CRP. Table 2 provides a detailed overview of the univariate analysis outcomes. Significant variables (*P* < 0.05) were included in a multivariable logistic regression model. Table 2 displays four factors independently linked to AFDAS after adjustments: advanced age (age ≥65 vs. <65 years: OR = 2.14, 95% CI: 1.44–3.16, *P* < 0.001), prior CHD history (OR = 1.88, 95% CI: 1.22–2.92, *P* = 0.005), elevated CRP levels (OR = 1.02, 95% CI: 1.01–1.02, *P* < 0.001), and increased TG levels (OR = 0.76, 95% CI: 0.61–0.96, *P* = 0.020). Figure 2 illustrates the forest plot of the multivariable logistic regression.

**Table 2:** The results of univariate and multivariate logistic regression.

**Variables**	**Univariate logistic regression**		**Multivariate logistic regression**	
	***P***	**OR (95% CI)**	***P***	**OR (95% CI)**
Age (<65 or ≥65)	**<0**.**001**	2.48 (2.07–2.97)	**<0**.**001**	2.14 (1.44–3.16)
Female/Male	**0**.**009**	1.25 (1.06–1.48)	0.156	1.31 (0.90–1.91)
BMI	**0**.**040**	0.97 (0.95–0.999)	0.716	1.01 (0.97–1.05)
Hypertension	0.948	1.01 (0.85–1.20)	–	–
Diabetes mellitus	0.642	0.95 (0.77–1.18)	–	–
Prior stroke or TIA	0.750	0.97 (0.81–1.16)	–	–
Coronary heart disease	**<0**.**001**	2.68 (2.21–3.25)	**0**.**005**	1.88 (1.22–2.92)
Heart failure	**0**.**050**	2.48 (1.00–6.15)	–	–
Peripheral Disease	0.1485	0.65 (0.37–1.16)	–	–
Current smoking	**0**.**002**	0.74 (0.61–0.90)	0.254	0.76 (0.47–1.21)
HbA1C	**0**.**021**	0.91 (0.85–0.99)	0.205	0.96 (0.80–1.05)
CRP	**< 0.001**	1.01 (1.01–1.02)	**<0**.**001**	1.02 (1.01–1.02)
TG	**< 0.001**	0.75 (0.67–0.83)	**0**.**020**	0.76 (0.61–0.96)
HDL	0.874	1.02 (0.82–1.27)	–	–
LDL	**<0**.**001**	0.79 (0.72–0.87)	0.990	0.999 (0.82–1.22)

Bold represents significant values where the *p*-value is less than 0.05; BMI, body mass index; CI: confidence intervals; CRP, c-reactive protein; HDL, high density protein; LDL, low density protein; OR: odds ratios; TIA, transient ischemic attack; TC, total cholesterol; TG, triglyceride; HbA1C, Glycated Hemoglobin A1C.

### 3.3 Association of AFDAS with recurrent stroke

**Figure 3:**
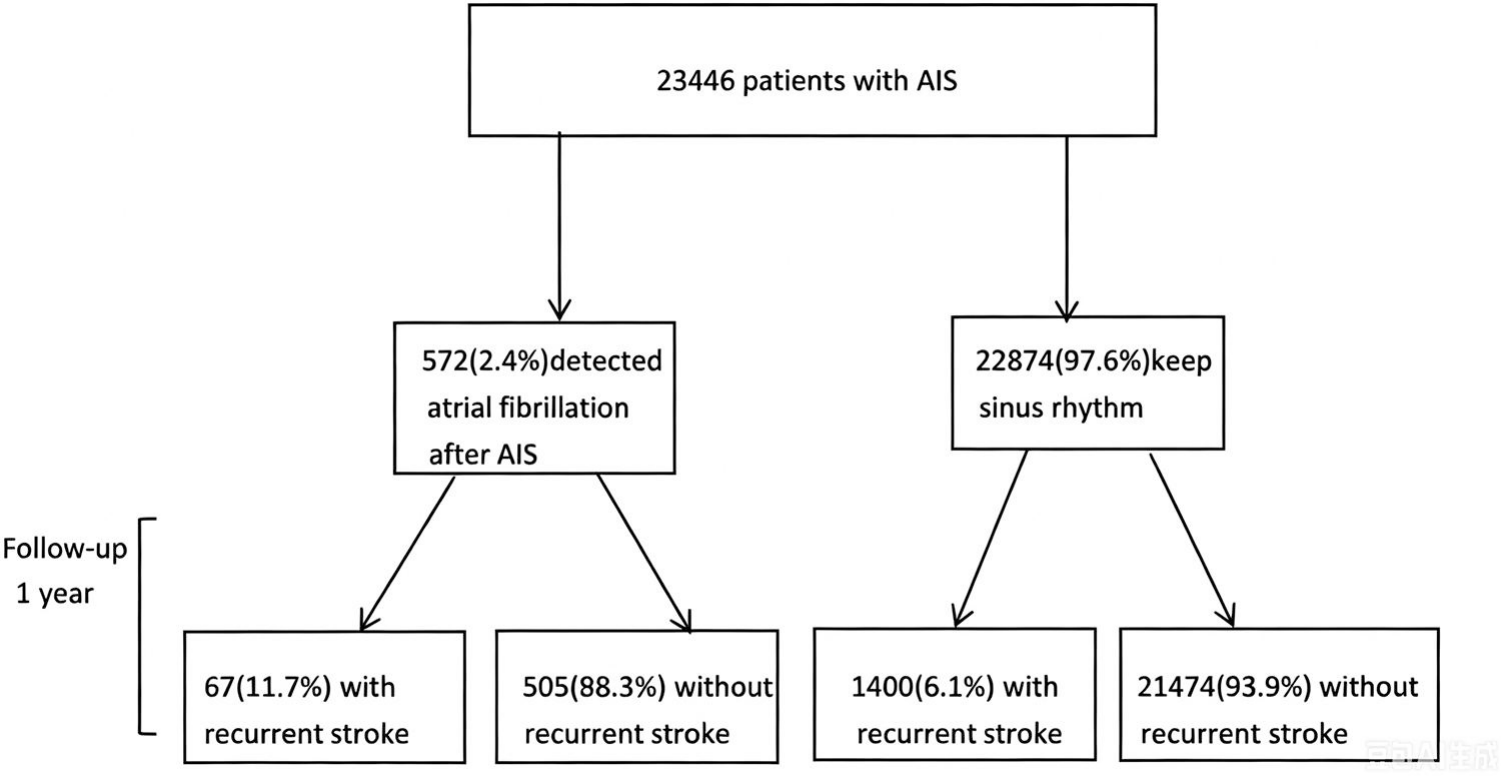
1-Year recurrent stroke (AFDAS vs. Sinus Rhythm). Bar chart comparing the 1-year stroke recurrence rate between patients with atrial fibrillation detected after stroke (AFDAS, 11.7%) and those with sinus rhythm (6.1%), showing a significantly higher recurrence risk in the AFDAS group.

During the one-year follow-up, mortality was 22.7% in patients with AFDAS and 7.1% in patients with SR (*P* < 0.001). Recurrent stroke occurred in 1,467 patients (6.3% of the entire cohort). Recurrent stroke occurred significantly more frequently in the AFDAS group than in the SR group [67/572 [11.7%] vs. 1,400/22,874 [6.1%], *P* < 0.001]. The distribution of recurrent stroke cases between groups is illustrated in Figure 3. Baseline clinical characteristics were compared between patients with and without recurrent ischemic stroke (Table 3). The prevalence of AFDAS was significantly higher in patients with recurrent stroke than in those without recurrence (4.6% vs. 2.3%, *P* < 0.001). After adjustment for traditional risk factors, including age, vascular comorbidities, and long-term medication status, AFDAS remained an independent risk factor for 1-year ischemic stroke recurrence (Table 4).

**Table 3:** Baseline characteristics of patients with recurrent stroke.

Risk factors	Recurrent stroke (*n* = 1,467)	Non-recurrent stroke (*n* = 21,979)	P
AFDAS, *n* (%)	67 * (4.6)	505 (2.3)	**<0**.**001**
Age, mean ± SD	67.4 ± 11.6	63.2 ± 10.1	**<0**.**001**
Female, *n* (%)	575 (39.2)	8,035 (36.5)	**0**.**028**
Hypertension, *n* (%)	1,084 (73.9)	14,052 (63.9)	**<0**.**001**
Diabetes mellitus, *n* (%)	367 (25.0)	4,031 (18.3)	**<0**.**001**
Current smoking, *n* (%)	350 (23.9)	6,537 (29.7)	**<0**.**001**
Prior stroke or TIA, *n* (%)	669 (45.6)	6,483 (29.4)	**<0**.**001**
Coronary heart disease, *n* (%)	239 (16.3)	2,413 (11.0)	**<0**.**001**
Heart failure, *n* (%)	13 (0.9)	73 (0.3)	**<0**.**001**
Peripheral disease, *n* (%)	57 (3.9)	681 (3.1)	**<0**.**001**
Drugs used at least 6 months			
Antiplatelet, *n* (%)	480 (32.7)	18,769 (85.3)	**<0**.**001**
Anticoagulation, *n* (%)	6 (0.4)	556 (2.5)	**<0**.**001**
Statins, *n* (%)	188 (12.8)	12,051 (54.8)	**<0**.**001**
Hypoglycemic, *n* (%)	170 (12.8)	4,847 (22.1)	**<0**.**001**

Bold represents significant values where the *p*-value is less than 0.05; AFDAS, atrial fibrillation detected after stroke; TIA, transient ischemic attack.

**Table 4:** Multivariate logistic regression results of AFDAS for 1-year recurrent ischemic stroke.

**Adjusted AFDAS**	**OR (95%CI)**	**P**
Model 1	1.82 (1.39–2.38)	**<0**.**001**
Model 2	1.68 (1.27–2.22)	**<0**.**001**
**Full multivariate-adjusted results of Model 2**		
**Variables**		
Age (per 1-year increase)	1.03 (1.02–1.04)	**<0**.**001**
Female	1.07 (0.97–1.17)	0.164
Hypertension	1.31 (1.18–1.44)	**<0**.**001**
Diabetes mellitus	1.21 (1.07–1.37)	**0**.**002**
Current smoking	0.81 (0.71–0.91)	**0**.**001**
Prior stroke or TIA	1.74 (1.57–1.93)	**<0**.**001**
Coronary heart disease	1.32 (1.14–1.53)	**<0**.**001**
Heart failure	1.86 (1.02–3.38)	**0**.**043**
Peripheral artery disease	1.11 (0.83–1.47)	0.468
Antiplatelet agents	0.14 (0.13–0.16)	**<0**.**001**
Statins	0.17 (0.14–0.20)	**<0**.**001**
Hypoglycemic agents	0.69 (0.59–0.82)	**<0**.**001**
Anticoagulants	0.23 (0.10–0.52)	**<0**.**001**

Model 1 (Adjusted for age, sex, hypertension, diabetes mellitus, prior stroke or TIA, coronary heart disease, heart failure, peripheral artery disease); Model 2 (Model 1 + further adjusted for antiplatelet agents, anticoagulants, statins, hypoglycemic agents); OR: odds ratios; CI: confidence intervals.

Bold represents significant values where the P-value is less than 0.05.

## 4 Discussion

Previous research has indicated that implantable event recorders or long-term cardiac monitoring can add AF detection in unselected stroke patients; however, the high cost and operational complexity of these devices limit their widespread application. Therefore, identifying high-risk populations for AFDAS through accessible clinical indicators is of great clinical significance. Our results showed that older age, a history of CHD, elevated CRP and lower TG levels at admission are associated with AFDAS. Notably, higher TG was identified as a protective factor for AFDAS, which contradicts the conventional understanding of TG as a cardiovascular risk factor and thus requires in-depth discussion.

In patients with AF-associated ischemic stroke, accumulating evidence demonstrates a higher recurrence risk in those with known pre-stroke AF than in those with AFDAS (10). This outcome provides empirical support for the proposition that AFDAS might be a distinct clinical phenotype rather than a variant of established AF, which is more likely to have a cardiogenic origin. It is hypothesized that AFDAS partially results from neurogenic, stroke-induced autonomic cardiac changes. The autonomic nervous system (ANS) is crucial in regulating cardiac arrhythmias. There is evidence that the onset of AF is significantly influenced by extrinsic cardiac ANS (17, 18). The findings of our investigation may then be explained by the fact that patients with coronary heart disease and other cardiac diseases are more susceptible to AF due to abnormal autonomic nerve regulation. Another explanation is that the etiology of AFDAS is heterogenic, involving patients with undetected pre-existent AF before stroke onset, as known AF is likely to be combined with CHD (19–21).

In our study, an elevated CRP level is a risk factor of AFDAS, even after adjusting for conventional risk factors, including CHD (22). This observed association has multiple interpretations. On one hand, elevated CRP may simply reflect greater stroke severity (e.g., larger infarct size), which itself increases the likelihood of AFDAS. On the other hand, CRP is a sensitive systemic marker of inflammation and tissue damage, and the acute inflammatory state triggered by a stroke may precipitate AF through neurogenic and autonomic dysregulation or promote atrial structural remodeling or electrical conduction heterogeneity (21, 23, 24). However, baseline CRP was measured at hospital admission, which may not fully reflect the peak inflammatory response after acute stroke, as CRP typically peaks at 36–72 h after stroke onset. And unrecognized concomitant infection may affect circulating CRP concentrations, but information on concurrent acute or chronic infection during admission was not available in the registry database. Therefore, the prognostic value of CRP in the present study should be interpreted with these measurement limitations in mind. Furthermore, the extent to which the observed CRP-AFDAS association represents an independent inflammatory pathway vs. being confounded by stroke severity remains to be clarified in future studies.

A striking finding of this study is that TG levels act as a protective factor of AFDAS, despite the well-established role of elevated TG in increasing the risk of cardiovascular diseases (25). TG may exert a context-dependent effect on inflammatory responses, and lower TG levels might attenuate inflammatory infiltration in atrial tissue, a key driver of AF (11, 26). However, the finding warrants cautious interpretation in this study population. The higher CHD prevalence in the AFDAS group suggests greater use of lipid-lowering therapy, which may decrease the TG level. Additionally, age-related malnutrition and frailty may lower TG levels independently. Thus, whether lower TG directly contributes to AFDAS or merely reflects treatment effects or reverse causation remains unclear. Future studies should further confirm this observation in independent cohorts.

The prognostic significance of AFDAS remains controversial in existing literature. Due to differences in mechanisms and clinical manifestations, AF-induced cardioembolic stroke typically presents with large, multiple, or bilateral cerebral infarcts, leading to greater severity and permanent disability (27, 28). In our study, by excluding patients with known AF before stroke, we found that AFDAS patients had a significantly higher 1-year stroke recurrence rate (11.7%) than SR patients (6.1%, *P* < 0.001)—a finding inconsistent with Sposato's retrospective study, which reported a 6.6% recurrence rate in AFDAS patients (29). After adjustment for comorbidities and anticoagulants, AFDAS is still the risk factor of stroke recurrence. This discrepancy is likely attributable to the heterogeneity of AFDAS, which encompasses cardiogenic, neurogenic, or mixed-type AF. Observational studies struggle to standardize the proportion of these subtypes, leading to variable results. Our study further identifies that AFDAS patients with recurrent stroke are more likely to have comorbidities such as CHD and prior stroke/TIA. These findings suggest that cardiovascular risk factors and comorbidities not only contribute to the development of AFDAS but also further amplify the recurrence risk, emphasizing the need for comprehensive management of comorbid conditions in AFDAS patients. However, the prognostic impact of atrial fibrillation needs deep discussion. This study is a secondary analysis of the CNSR-II registry, an observational cohort based on real-world clinical practice. ECG examinations and in-hospital cardiac monitoring were performed according to routine clinical workflows rather than standardized prolonged monitoring (such as ≥72-hour Holter). Consequently, numerous patients with paroxysmal atrial fibrillation may be misclassified into the sinus rhythm group. In addition, patients in the AFDAS group were older and carried more comorbidities, which likely prolonged their cardiac monitoring duration and increased the likelihood of incidental AF detection. This may lead to weakly biased statistical comparisons of AFDAS and SR.

Several limitations should be acknowledged in the present study. First, the main limitation is that patients in the CNSR-II study did not undergo 72-hour or longer Holter monitoring, which could increase the detection of AF compared with routine clinical follow-up. Additionally, although patients with a documented AF history were excluded at enrollment, occult cardioembolic sources could not be completely ruled out in this real-world cohort. The AFDAS phenotype specifically captures previously unrecognized AF and hidden cardioembolic mechanisms, which should be fully considered when interpreting the study results. Second, several important clinical factors linked to AFDAS risk—including TOAST subtype and echocardiographic markers, such as left atrial diameter—were unavailable for adjustment in our multivariate models, which may limit the accuracy of independent predictor identification. The study intentionally used simple, readily available indicators for routine practice; thus, NIHSS was not included in the primary regression. However, unadjusted baseline stroke severity may introduce residual confounding and bias the associations between CRP, AFDAS, and stroke recurrence.

Third, stroke recurrence was adjudicated based on telephone follow-up without systematic imaging confirmation, making it difficult to fully distinguish true recurrent ischemic stroke from early neurological deterioration or progression of the index event, which may introduce misclassification bias. Moreover, the rate of long-term oral anticoagulation was lower in AFDAS patients during follow-up, which could increase recurrence risk. As this is an observational registry study and data were collected in 2012–2013, we are unable to precisely analyze the underlying reasons for such inadequate anticoagulant prescriptions. Notably, improved adherence to anticoagulant therapy in contemporary clinical practice may alter the observed associations and final results of this study. Finally, several statistical limitations should be noted. Multicollinearity diagnostics (VIF/tolerance) were not performed due to database constraints. The missing rate of core laboratory variables was approximately 18% in the first regression model; for the second model, precise missing data were unavailable (estimated <20%). The complete-case analysis applied to multivariable models, with missing rates up to 20%, may have introduced selection bias and compromised the representativeness and robustness of our findings. Given these limitations, our findings should be interpreted with caution and warrant further validation in larger, longer-term studies to confirm their robustness and generalizability.

## 5 Conclusion

In conclusion, these findings support the use of readily available clinical and laboratory parameters for early risk stratification of AFDAS and underscore the need for intensified secondary prevention in this high-risk population. Further prospective studies with standardized prolonged cardiac monitoring are warranted to validate these associations.

## Data Availability

The original contributions presented in the study are included in the article/Supplementary Material, further inquiries can be directed to the corresponding author.

## References

[1] Feigin VL Owolabi MO. Pragmatic solutions to reduce the global burden of stroke: a world stroke organization-lancet neurology commission. Lancet Neurol. (2023) 22(12):1160–206. 10.1016/S1474-4422(23)00277-6 37827183 PMC10715732

[2] Zhao H Lu S Jie Y Chao W Zhu W Huang D. Comprehensive analysis of the ischemic stroke burden at global, regional, and national levels (1990–2021): trends, influencing factors, and future projections. Front Neurol. (2025) 16:1492691. 10.3389/fneur.2025.1492691 40177409 PMC11961430

[3] Romero JR Wolf PA. Epidemiology of stroke: legacy of the framingham heart study. Glob Heart. (2013) 8(1):67–75. 10.1016/j.gheart.2012.12.007 23527318 PMC3601756

[4] Liao J Khalid Z Scallan C Morillo C O'Donnell M. Noninvasive cardiac monitoring for detecting paroxysmal atrial fibrillation or flutter after acute ischemic stroke: a systematic review. Stroke. (2007) 38(11):2935–40. 10.1161/STROKEAHA.106.478685 17901394

[5] Lazzaro MA Krishnan K Prabhakaran S. Detection of atrial fibrillation with concurrent holter monitoring and continuous cardiac telemetry following ischemic stroke and transient ischemic attack. J Stroke Cerebrovasc Dis. (2012) 21(2):89–93. 10.1016/j.jstrokecerebrovasdis.2010.05.006 20656504

[6] Sposato LA Cipriano LE Saposnik G Ruíz Vargas E Riccio PM Hachinski V. Diagnosis of atrial fibrillation after stroke and transient ischaemic attack: a systematic review and meta-analysis. Lancet Neurol. (2015) 14(4):377–87. 10.1016/S1474-4422(15)70027-X 25748102

[7] Lip GY Hunter TD Quiroz ME Ziegler PD Turakhia MP. Atrial fibrillation diagnosis timing, ambulatory ECG monitoring utilization, and risk of recurrent stroke. Circ Cardiovasc Qual Outcomes. (2017) 10(1):e002864. 10.1161/CIRCOUTCOMES.116.002864 28096204

[8] Yang XM Rao ZZ Gu HQ Zhao XQ Wang CJ Liu LP. Atrial fibrillation known before or detected after stroke share similar risk of ischemic stroke recurrence and death. Stroke. (2019) 50(5):1124–9. 10.1161/STROKEAHA.118.024176 31009353

[9] Bhatla A Borovskiy Y Katz R Hyman MC Patel PJ Arkles J. Stroke, timing of atrial fibrillation diagnosis, and risk of death. Neurology. (2021) 96(12):e1655–e62. 10.1212/WNL.0000000000011633 33536273

[10] Fridman S Jimenez-Ruiz A Vargas-Gonzalez JC Sposato LA. Differences between atrial fibrillation detected before and after stroke and TIA: a systematic review and meta-analysis. Cerebrovasc Diseases (Basel, Switzerland). (2022) 51(2):152–7. 10.1159/000520101 34844239

[11] Kleindorfer DO Towfighi A Chaturvedi S Cockroft KM Gutierrez J Lombardi-Hill D. 2021 Guideline for the prevention of stroke in patients with stroke and transient ischemic attack: a guideline from the American Heart Association/American stroke association. Stroke. (2021) 52(7):e364–467. 10.1161/STR.0000000000000375 34024117

[12] Ntaios G. Embolic stroke of undetermined source: jACC review topic of the week. J Am Coll Cardiol. (2020) 75(3):333–40. 10.1016/j.jacc.2019.11.024 31976872

[13] Uphaus T Weber-Krüger M Grond M Toenges G Jahn-Eimermacher A Jauss M. Development and validation of a score to detect paroxysmal atrial fibrillation after stroke. Neurology. (2019) 92(2):e115–e24. 10.1212/WNL.0000000000006727 30530796

[14] Yang X Li Z Zhao X Wang C Liu L Wang C. Use of warfarin at discharge among acute ischemic stroke patients with nonvalvular atrial fibrillation in China. Stroke. (2016) 47(2):464–70. 10.1161/STROKEAHA.115.011833 26696643

[15] Li Z Wang C Zhao X Liu L Wang C Li H. Substantial progress yet significant opportunity for improvement in stroke care in China. Stroke. (2016) 47(11):2843–9. 10.1161/STROKEAHA.116.014143 27758941

[16] Zong L Wang X Li Z Zhao X Liu L Li H. Alkaline phosphatase and outcomes in patients with preserved renal function: results from China national stroke registry. Stroke. (2018) 49(5):1176–82. 10.1161/STROKEAHA.118.020237 29669879

[17] Shen MJ Zipes DP. Role of the autonomic nervous system in modulating cardiac arrhythmias. Circ Res. (2014) 114(6):1004–21. 10.1161/CIRCRESAHA.113.302549 24625726

[18] Chen PS Chen LS Fishbein MC Lin SF Nattel S. Role of the autonomic nervous system in atrial fibrillation: pathophysiology and therapy. Circ Res. (2014) 114(9):1500–15. 10.1161/CIRCRESAHA.114.303772 24763467 PMC4043633

[19] Wang Y Qian Y Smerin D Zhang S Zhao Q Xiong X. Newly detected atrial fibrillation after acute stroke: a narrative review of causes and implications. Cardiology. (2019) 144(3-4):112–21. 10.1159/000502971 31600748

[20] Cerasuolo JO Cipriano LE Sposato LA. The complexity of atrial fibrillation newly diagnosed after ischemic stroke and transient ischemic attack: advances and uncertainties. Curr Opin Neurol. (2017) 30(1):28–37. 10.1097/WCO.0000000000000410 27984303 PMC5321114

[21] Sposato LA Hilz MJ Aspberg S Murthy SB Bahit MC Hsieh CY. Post-Stroke cardiovascular complications and neurogenic cardiac injury: jACC state-of-the-art review. J Am Coll Cardiol. (2020) 76(23):2768–85. 10.1016/j.jacc.2020.10.009 33272372

[22] Aviles RJ Martin DO Apperson-Hansen C Houghtaling PL Rautaharju P Kronmal RA. Inflammation as a risk factor for atrial fibrillation. Circulation. (2003) 108(24):3006–10. 10.1161/01.CIR.0000103131.70301.4F 14623805

[23] Hu YF Chen YJ Lin YJ Chen SA. Inflammation and the pathogenesis of atrial fibrillation. Nat Rev Cardiol. (2015) 12(4):230–43. 10.1038/nrcardio.2015.2 25622848

[24] Kallergis EM Manios EG Kanoupakis EM Mavrakis HE Kolyvaki SG Lyrarakis GM. The role of the post-cardioversion time course of hs-CRP levels in clarifying the relationship between inflammation and persistence of atrial fibrillation. Heart. (2008) 94(2):200–4. 10.1136/hrt.2006.108688 17575330

[25] Chapman MJ Ginsberg HN Amarenco P Andreotti F Boren J Catapano AL. Triglyceride-rich lipoproteins and high-density lipoprotein cholesterol in patients at high risk of cardiovascular disease: evidence and guidance for management. Eur Heart J. (2011) 32(11):1345–61. 10.1093/eurheartj/ehr112 21531743 PMC3105250

[26] Alonso A Yin X Roetker NS Magnani JW Kronmal RA Ellinor PT. Blood lipids and the incidence of atrial fibrillation: the multi-ethnic study of atherosclerosis and the framingham heart study. J Am Heart Assoc. (2014) 3(5):e001211. 10.1161/JAHA.114.001211 25292185 PMC4323837

[27] Hannon N Sheehan O Kelly L Marnane M Merwick A Moore A. Stroke associated with atrial fibrillation—incidence and early outcomes in the north Dublin population stroke study. Cerebrovascular Diseases. (2010) 29(1):43–9. 10.1159/000255973 19893311 PMC2914401

[28] Min J Farooq MU. Detecting nonvalvular atrial fibrillation and anticoagulant therapy in cardioembolic ischemic stroke. Postgrad Med. (2016) 128(6):620–8. 10.1080/00325481.2016.1195236 27263867

[29] Sposato LA Cerasuolo JO Cipriano LE Fang J Fridman S Paquet M. Atrial fibrillation detected after stroke is related to a low risk of ischemic stroke recurrence. Neurology. (2018) 90(11):e924–e31. 10.1212/WNL.0000000000005126 29444969

